# 利用在线加压溶剂提取-超高效液相色谱-离子阱-飞行时间-质谱法定性分析片仔癀化学成分组

**DOI:** 10.3724/SP.J.1123.2020.10011

**Published:** 2021-05-08

**Authors:** Wei LI, Zhenzhen JIANG, Han LI, Pengfei TU, Qingqing SONG, Juan YU, Yuelin SONG

**Affiliations:** 1.北京中医药大学中药学院, 中药现代研究中心, 北京 100029; 1. Modern Research Center for Traditional Chinese Medicine, School of Chinese Materia Medica, Beijing University of Chinese Medicine, Beijing 100029, China; 2.漳州片仔癀药业股份有限公司, 福建 漳州 363000; 2. Zhangzhou Pien-Tze-Huang Pharmaceutical Co., Ltd., Zhangzhou 363000, China

**Keywords:** 在线加压溶剂提取, 片仔癀, 化学成分组, 质谱裂解途径, 来源归属, online pressurized liquid extraction, Pien-Tze-Huang, chemome profiling, mass fragmentation pathways, source attribution

## Abstract

片仔癀(Pien-Tze-Huang)是由三七、牛黄、蛇胆、麝香等名贵中药经加工精制而成的中药制剂。片仔癀中的三七皂苷、胆汁酸以及麝香酮等主要化学成分已被深入研究,然而其全方化学成分组成尚未被整体阐明。该文建立了在线加压溶剂提取-超高效液相色谱-离子阱-飞行时间质谱(online PLE-UHPLC-IT-TOF-MS)法,快速、直接分析片仔癀化学成分组。将少量片仔癀粉末(0.3 mg)均匀地平铺于预柱芯尾端,之后用正相硅胶填充预柱芯,滤膜密封后构成提取池。提取池装入预柱套后置于70 ℃的柱温箱内,连接于UHPLC-IT-TOF-MS分析系统。通过引入一个二位六通电子阀,将整个分析过程自动在提取相和洗脱相间切换。提取相用时3 min,以0.1%(v/v)甲酸水为提取溶剂,流速为0.2 mL/min;洗脱相以0.1%(v/v)甲酸水和乙腈为流动相进行梯度洗脱,IT-TOF-MS检测。通过与对照品、相关文献和自建中药数据库对照,并总结相关质谱裂解规律,从片仔癀中共检测到73个化学成分,初步鉴定并归属了其中71个,36个来源于三七,15个来源于蛇胆,9个来源于牛黄,11个可能来源于牛黄与蛇胆,另有2个结构无法确定。该研究深入解析了片仔癀的化学成分组,为其质量分析提供了丰富的信息。同时,该文构建的online PLE-UHPLC-IT-TOF-MS分析系统为中药复杂体系快速、直接分析提供了可靠的工具。

## Abstract (en)

Pien-Tze-Huang is one of the most famous traditional Chinese medicine prescriptions and consists of several precious medicinal materials, such as Notoginseng Radix et Rhizoma, Bovis Calculus, Snake Gall, and Moschus. However, its formula has not been completely revealed. It is mainly applied for the treatment of acute and chronic viral hepatitis, carbuncle, and boils caused by blood stasis, unknown swelling, bruises, and various inflammation disorders. The chemical composition of Pien-Tze-Huang is extremely complicated. Thus far, extensive attention has been paid to the principal chemical families in Pien-Tze-Huang, such as ginsenosides, bile acids, and muscone derivatives. Comprehensive chemical profiling, although of immense importance for systematic quality control, has not been achieved. Therefore, we configured a platform, namely online pressurized liquid extraction-ultra-high-performance liquid chromatography-ion trap-time-of-flight mass spectrometry (online PLE-UHPLC-IT-TOF-MS), to characterize the chemical profile of Pien-Tze-Huang in detail as well as to conduct source attribution, aiming to clarify the chemome of Pien-Tze-Huang and to provide a reliable method for quality assessment. A sub-microgram amount of Pien-Tze-Huang powder (0.3 mg) was placed in a hollow guard column, which was subsequently filled with clear silica gel. Filter membranes were used to seal the extraction vessel. The vessel was then placed in an adapted guard column holder and maintained in a thermal column oven (70 ℃). Metal tubing was used to connect the outlet of the guard column holder to the mass spectrometer. The extraction phase was maintained for 3 min by employing 0.1%(v/v) formic acid aqueous solution as the extraction solvent with a flow rate of 0.2 mL/min. Moreover, a six-port two-position electronic valve was introduced to automatically switch the system from extraction to elution phases. Within the elution phase, 0.1%(v/v) formic acid aqueous solution and acetonitrile composed the mobile phase, and the extracts were eluted with a gradient program. Because of the elevated temperature and pressure, the physical and chemical properties of water, especially polarity and solubility, were modified. Therefore, warm water could be an eligible green solvent to achieve wide polarity-spanned extraction. In addition, IT-TOF-MS was employed to acquire tandem mass spectrometry information. The mass fragmentation pathways of saponins and bile acids were carefully studied. Finally, according to authentic compounds, mass fragmentation pathways, reference information in the literature, and accessible databanks, a total of 73 signals were observed from Pien-Tze-Huang, of which 71 components were tentatively identified and assigned. Among them, 36 were from Notoginseng Radix et Rhizoma, 15 from Snake Gall, and 9 from Bovis Calculus, while the occurrences of the other 11 components were synergistically contributed by both Bovis Calculus and Snake Gall, through retrieving the in-house chemical database that was built by considering all accessible chemical information from Notoginseng Radix et Rhizoma, Bovis Calculus, Snake Gall, and Moschus. The other two compounds were assigned as unknown compounds. However, none of the components were assigned to Moschus because they mainly contained hydrophobic compounds, such as cycloketones, cholesterol, and sterols, among others, and it was difficult to detect them with the current measurement program. The extraction efficiency of online PLE was assessed by comparing it with the efficiency obtained from ultrasonication at the same time. According to base peak ion current chromatograms (BPCs) and mass spectrometry information, the efficiency of online PLE was greater than that of ultrasonic extraction, even through direct analysis. Online PLE-UHPLC-IT-TOF-MS is not only a tool fit for the concept of green analytical chemistry, but also a reliable analytical pipeline for the direct characterisation of other complicated matrixes. Above all, this study clarified the chemome of Pien-Tze-Huang and provided meaningful information for the quality control of this famous TCM prescription.

片仔癀(Pien-Tze-Huang)具有清热解毒、消肿止痛、凉血化瘀之功效^[1]^,可用于热毒血瘀所致急慢性肝炎,痈疽疔疮,跌打损伤、无名肿痛及各种炎症。其药用历史悠久,为国家一级中药保护制剂,目前公开组方包括三七、牛黄、蛇胆、麝香4味名贵中药。现代药理研究表明片仔癀具有治疗急慢性肝炎、溃疡、外伤、脑保护、抗肿瘤等作用^[2,3,4,5]^。片仔癀中三七皂苷、胆汁酸以及麝香酮等主要化学成分已被系统研究,但对片仔癀复方的化学成分进行全面表征和解析的相关研究较少。中药复方是中医治病的主要临床用药形式,其化学成分并不是各单味中药化学成分的相加,需要全面、系统地阐明复方化学成分组,进而为其质量控制标准及药效研究提供可靠的依据。

常用的提取方法如浸渍、回流和超声等费时费力,还需要大量的溶剂和药材,提取效率低。本课题组前期建立了在线加压溶剂提取方法(online pressurized liquid extraction, online PLE),采用水作为绿色提取溶剂,通过升温和加压提高水对药材中化学成分的溶解和提取能力,实现了快速、高效提取。Online PLE模块可以连接于液相色谱系统实现直接分析,有效地避免了不稳定化学成分在提取等制备过程中发生降解^[6]^。课题组利用该仪器平台定性、定量表征大小鼠粪便以及远志、肉苁蓉等中药的化学成分组成^[7,8,9]^。片仔癀含有的牛磺酸结合型胆汁酸由于酰胺键的存在易发生水解或氧化^[10]^,利用在线加压溶剂提取方法能够有效降低该类成分在提取时降解的可能性。并且,online PLE可以实现毫克级样品分析,特别适合贵重药材的化学成分组成表征。因此,本研究利用在线加压溶剂提取-超高效液相色谱-离子阱-飞行时间质谱(online PLE-UHPLC-IT-TOF-MS)对片仔癀化学成分组进行快速、全面分析。

## 1 实验部分

### 1.1 仪器、试剂与药材

岛津分析型超高效液相色谱仪,包含LC-20AD泵、CTO-20A柱温箱、SIL-20A自动进样器、SPD-M20A紫外检测器、DGU-20A脱气机、CBM-20A控制器和色谱工作站(日本岛津公司);岛津质谱仪IT-TOF-MS(日本岛津公司);万分之一电子天平(METTLER XS105型,瑞士梅特勒-托利多仪器有限公司);超声波清洗器(南京垒君达超声电子设备有限公司);超纯水净化系统(美国密理博有限公司);超速离心机(德国艾本德公司)。Phenomenex预柱芯(型号:AJ0-4287)和适配的Security Guard^TM^预柱套(型号:KJ0-4282,飞诺美公司)。预柱芯去除硅胶,用于填装片仔癀粉末。

质谱级甲醇、甲酸、乙腈购于美国赛默飞世尔公司,超纯水由实验室Milli-Q纯水系统制备。片仔癀(粒)购自北京金象大药房医药连锁有限责任公司,批号为1908096。

对照品人参皂苷Rc、Rd、Re、Rf、Rg2、Rg3、Rh1、Rh2、Ro、F1、F2、拟人参皂苷F11、三七皂苷R1均由北京大学天然药物及仿生药物国家重点实验室提供,经HPLC-二极管阵列检测器-IT-TOF-MS检测,纯度均大于95%。胆酸(CA)、熊去氧胆酸(UDCA)、猪去氧胆酸(HDCA)、鹅去氧胆酸(CDCA)、甘氨胆酸(GCA)、牛磺鹅去氧胆酸(TCDCA)均购自上海源叶生物科技有限公司,经HPLC-蒸发光散射检测器检测,纯度均大于98%。

### 1.2 Online PLE-UHPLC-IT-TOF-MS分析方法的建立

1.2.1 Online PLE提取及色谱洗脱条件

提取 取片仔癀样品一粒,用研钵研磨成细粉,精密称取0.3 mg,置于空预柱芯的尾端,用正相硅胶填充预柱芯,构成提取池。将提取池装入Phenomenex Security Guard^TM^预柱套中,置于70 ℃的柱温箱内。构建online PLE-UHPLC-IT-TOF-MS检测系统,0.1%(v/v)甲酸水为提取溶剂,流速为0.2 mL/min,提取3 min。通过引入一个二位六通阀,将在线加压提取模块直接连入液相色谱系统实现直接分析。当二位六通阀处于1-2位连接,通过自动进样2 μL 0.1%(v/v)甲酸水触发提取阶段,系统提取流路装置图见图1a。

**图 1:**
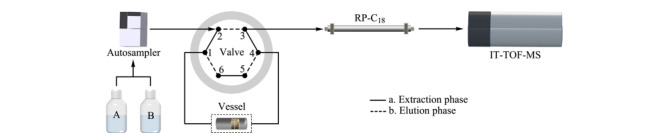
在线加压溶剂提取-超高效液相色谱-离子阱-飞行时间质谱系统装置图

洗脱及分析 采用Waters HSS T3色谱柱(100 mm×2.1 mm, 1.8 μm)。流动相组成为0.1%(v/v)甲酸水(A)和乙腈(B)。梯度洗脱程序:0~3 min, 0%B; 3~18 min, 0%B~36%B; 18~28 min, 36%B~55%B; 28~41 min, 55%B~85%B; 41~43 min, 85%B~95%B; 43~43.01 min, 95%B~0%B; 43.01~48 min, 0%B,流速为0.2 mL/min。在提取3 min后,二位六通阀切换至1-6位,进入洗脱分析程序。

1.2.2 质谱条件

采用电喷雾离子源(ESI),负离子模式下MS^1^扫描范围:*m/z* 100~1500, MS^2^扫描范围:*m/z* 50~1500;碰撞诱导解离(CID)能量:90%。喷雾室电压:-3.5 kV;曲型脱溶剂管(CDL)温度:200 ℃;加热模块温度:200 ℃;干燥气电压:100 MPa;雾化气流速:1.5 L/min;检测器电压:1.5 kV。离子阱压力:3.0×10^-2^ Pa;碰撞室压力:2.0×10^-4^ Pa;各级质谱的重复次数为3,离子累积时间为30 ms。

### 1.3 对照品溶液制备与分析

精密称定各对照品适量,用甲醇溶解,配制成10 g/L的对照品储备液。精密吸取各对照品储备液适量,用甲醇稀释,制备成混合对照品溶液。预柱芯仅用正相硅胶填充,自动进样器吸取2 μL混合对照品溶液注入分析系统,按1.2节条件分析。

### 1.4 超声提取样品的制备与分析

1.4.1 供试品溶液的制备

精密称定约50 mg片仔癀粉末,用1 mL甲醇超声提取30 min后,室温12000 r/min离心10 min,用0.22 μm滤膜过滤上清液,取续滤液待用。

1.4.2 分析条件

实验装置与图1b相同,二位六通阀处于1-6位。梯度洗脱程序:0~3 min, 0%B~5%B; 3~18 min, 5%B~36%B; 18~48 min同1.2.1节梯度。色谱柱、流动相及流速等色谱条件与质谱条件同1.2.1节和1.2.2节。

### 1.5 化学信息数据库的建立

在中国知网、SCIFinder、Pubmed等数据库中广泛检索片仔癀4味单味药三七、牛黄、蛇胆和麝香的化学成分分离和分析文献,将三七皂苷、胆汁酸、麝香酮、甾体激素等化学类型的结构和质谱信息录入Microsoft Excel软件,自建化学成分数据库。

## 2 结果与讨论

### 2.1 提取条件的优化

为了增大在线加压溶剂提取方法的效率,优化所有化合物的色谱及质谱行为,本实验在课题组前期工作^[8]^的基础上,进一步对提取和色谱条件进行优化。随着提取流速的升高(0.1、0.2和0.3 mL/min),系统反压也会增加,当选择0.2 mL/min为提取流速时,能够满足片仔癀中化学成分全面提取所需的压力和提取效率;同时,随着提取温度的升高(50、60、70 ℃),水的黏度降低且溶出能力增强,提取效率增高,故最终选择70 ℃为提取温度;对提取时间(3、4和5 min)进行优化,发现3 min即可提取完全,且此时提取液体积并不会影响色谱洗脱时的峰形。由于本实验仅引入一个二位六通阀,故需要优化合适的流动相作为提取溶剂,首先对比了水、0.05%甲酸水、0.1%甲酸水对提取效率和色谱峰形的影响,0.1%甲酸水能够产生更好的色谱峰形、增强质谱响应;之后,考察了提高提取流动相中乙腈的体积分数为5%、10%、15%时的提取效果,结果表明大多数成分难以提取出来,色谱峰数量和响应显著降低,故选择0.1%甲酸水作为提取溶剂。最终,本实验选择0.1%甲酸水作为提取溶剂,提取流速、提取时间及提取温度分别为0.2 mL/min、3 min和70 ℃。片仔癀中化学成分类型丰富,为了各化学成分有较好的色谱保留,提高色谱峰容量,最终选择了耐纯水的Waters HSS T3色谱柱。

### 2.2 在线加压溶剂提取法与超声提取法的对比

在相同的液相色谱和质谱条件下,对比了在线加压溶剂提取法与超声提取法的提取效率。从二者的基峰色谱图(见图2)可以看出,两种方法检出的色谱峰具有一定的相似性,然而在线加压溶剂提取法(见图2a)的提取效率显著高于

**图 2:**
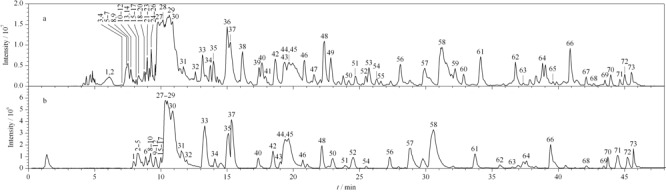
负离子模式下片仔癀的基峰离子流色谱图

甲醇超声提取。通过指认两张色谱图上的色谱峰,可以发现甲醇超声提取所得到的化学成分在在线加压溶剂提取样品中均有检出,且从色谱峰数量来看,在线加压溶剂提取仅需片仔癀0.3 mg即可提取出更多的化学成分,尤其是对于中等极性化学成分的提取效率明显高于超声提取(见图2b)。综上,在线加压溶剂提取方法能够高效、快速地提取中药化学成分。

### 2.3 片仔癀中皂苷类及胆酸类化合物的质谱裂解规律推导

由于麝香的化学成分主要为极性较小的大环酮及胆甾醇等化合物,难以在C_18_色谱柱上洗脱,且也未能检出麝香相关的化学成分。因此,本实验主要对片仔癀中三七、蛇胆与牛黄中的皂苷及胆酸类对照品的质谱行为进行分析,总结其裂解规律,用于片仔癀中化学成分的鉴定。

2.3.1 皂苷类化合物的鉴定

片仔癀中皂苷类化合物主要来源于三七,多为达玛烷型四环三萜,在C-3、C-6和C-20位连接有葡萄糖、鼠李糖等组成的糖链。按照苷元的不同,主要分为人参二醇型和人参三醇型人参皂苷。

以人参皂苷Rg3(ginsenoside Rg3, 65(图2中色谱峰号,下同))为例,阐述皂苷类化合物的质谱裂解规律及途径。负离子模式下,准分子离子峰为*m/z* 829.4822([M+HCOO]^-^),二级质谱图中检测到碎片离子主要为*m/z* 783.4747([M-H]^-^)、621.4249([M-H-glc]^-^)和459.3678([M-H-2glc]^-^)(见图3a),由于人参皂苷Rg3的C-3位连接一个两分子葡萄糖残基的糖链,推测其碎片离子分别由准分子离子丢失甲酸分子、一分子葡萄糖以及两分子葡萄糖产生,*m/z* 459.3678为其苷元特征碎片,人参皂苷Rg3的质谱裂解途径见图3b。化合物58、59与65具有相同的准分子离子峰,三者预测分子式均为C_42_H_72_O_13_,化合物65与59具有相同的二级碎片,推断为一组同分异构体。

**图 3:**
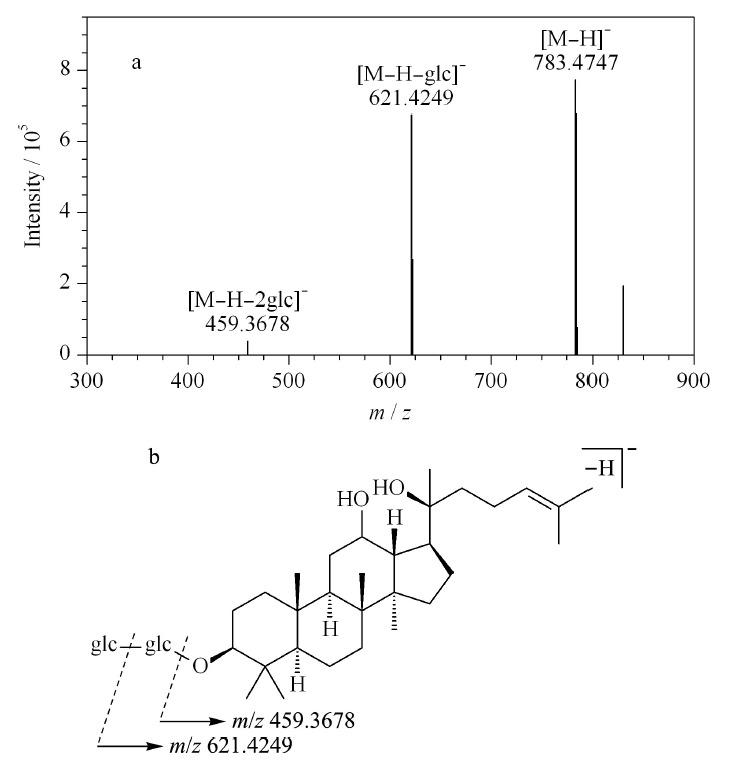
人参皂苷Rg3的(a)MS^2^图谱和(b)质谱裂解途径

结合文献^[11]^,在负离子模式下,皂苷类化合物一级质谱中易产生[M-H]^-^或[M+HCOO]^-^的准分子离子峰,其糖苷键断裂,会中性丢失葡萄糖残基(162 Da)、木糖残基(132 Da)、阿拉伯糖残基(132 Da)、鼠李糖残基(146 Da)和水(18 Da),从而形成苷元碎片离子,其中人参二醇型和人参三醇型人参皂苷元的碎片离子分别为*m/z* 459和*m/z* 475。

2.3.2 胆酸类化合物的鉴定

游离胆酸结构中存在羧基以及多个羟基,脱羧基反应以及失羟基为主要的裂解方式。有研究表明羟基位置不同其丢失水分子的能力也不同,且强弱顺序依次递减(7*α*>6*α*>12*α*>3*α*)^[12]^。当12位存在羟基时,会与24位羧基之间发生质子转移,进而得到[M-H-CH_2_O_2_]^-^碎片。以胆酸(cholic acid, CA, 55)为例,阐述游离型胆酸类化合物的质谱裂解规律。在负离子模式下,CA的准分子离子为*m/z* 407.2785([M-H]^-^), CA在C-3、C-7以及C-12位均有羟基取代,在二级质谱中能够看到丢失水分子以及羧基所生成的主要碎片离子为*m/z* 389.2666([M-H-H_2_O]^-^)、361.2698([M-H-CH_2_O_2_]^-^)、345.2771([M-H-H_2_O-CO_2_]^-^)、343.2618([M-H-H_2_O-CH_2_O_2_]^-^)和327.2652([M-H-2H_2_O-CO_2_]^-^)(见图4a),其质谱裂解途径见图4b。

**图 4:**
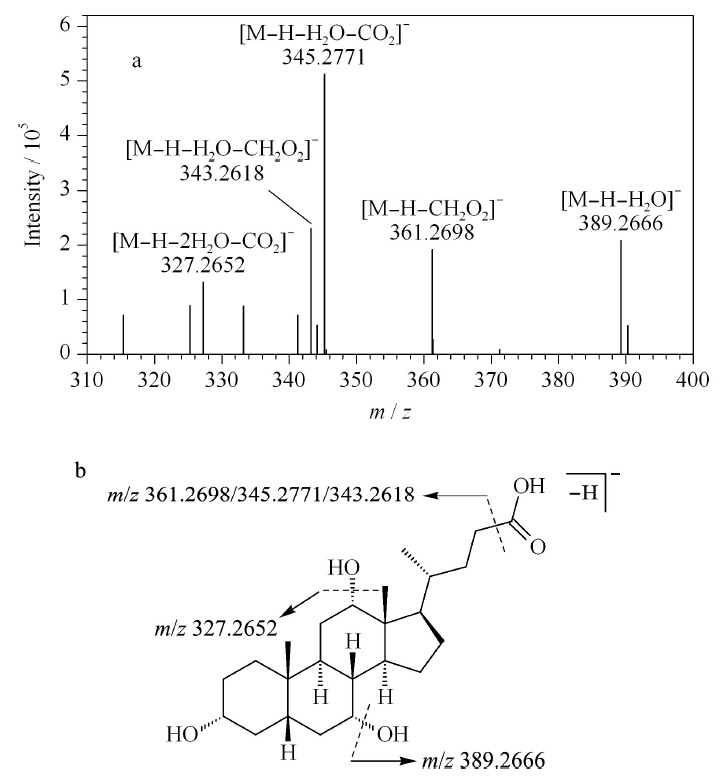
胆酸的(a)MS^2^图谱和(b)质谱裂解途径

以牛磺鹅去氧胆酸(taurochenodeoxycholic acid, TCDCA, 50)为例,阐述牛磺酸结合型胆酸的质谱裂解规律。在负离子模式下,TCDCA的准分子离子峰为*m/z* 498.2873([M-H]^-^),二级碎片离子主要为*m/z* 480.2806([M-H-H_2_O]^-^)和355.2612([M-H-H_2_O-C_2_H_6_NO_3_S]^-^)(见图5a)。TCDCA中C-24位羧基与牛磺酸基形成酰胺键易断裂,归属碎片离子分别为分子离子峰丢失水分子、牛磺酸残基产生,并未见母核碎裂的相关碎片离子,其质谱裂解途径见图5b。

**图 5:**
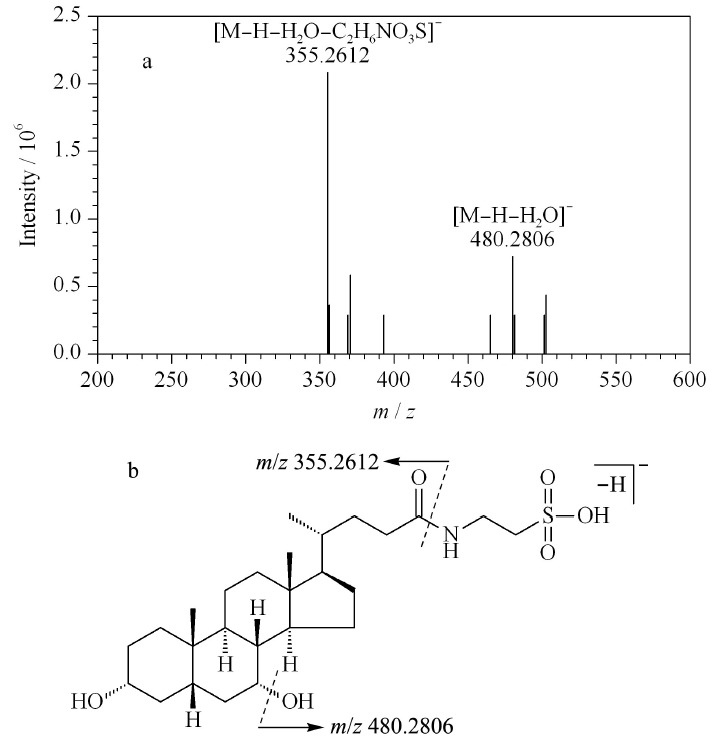
牛磺鹅去氧胆酸的(a)MS^2^图谱和(b)质谱裂解方式

以甘氨胆酸(glycocholic acid, 42)为例,阐述甘氨酸结合型胆酸的质谱裂解规律。在负离子模式下,准分子离子峰*m/z* 464.3001([M-H]^-^)。甘氨结合型胆酸是由胆酸的24位羧基经酰胺键与甘氨酸相连形成,酰胺键易发生断裂,同时,由于甘氨酸包括羧基,C-26位易发生脱羧失去44 Da(CO_2_)。故碎片离子归属为*m/z* 446.2895([M-H-H_2_O]^-^)、402.2987([M-H-H_2_O-CO_2_]^-^)、382.2730([M-H-2H_2_O-CH_2_O_2_]^-^)和353.2466([M-H-2H_2_O-C_2_H_4_NO_2_]^-^)(见图6a),推测准分子离子峰会丢失水分子、二氧化碳分子、甲酸分子以及甘氨酸残基生成相应的碎片离子,甘氨胆酸的质谱裂解途径见图6b。

**图 6:**
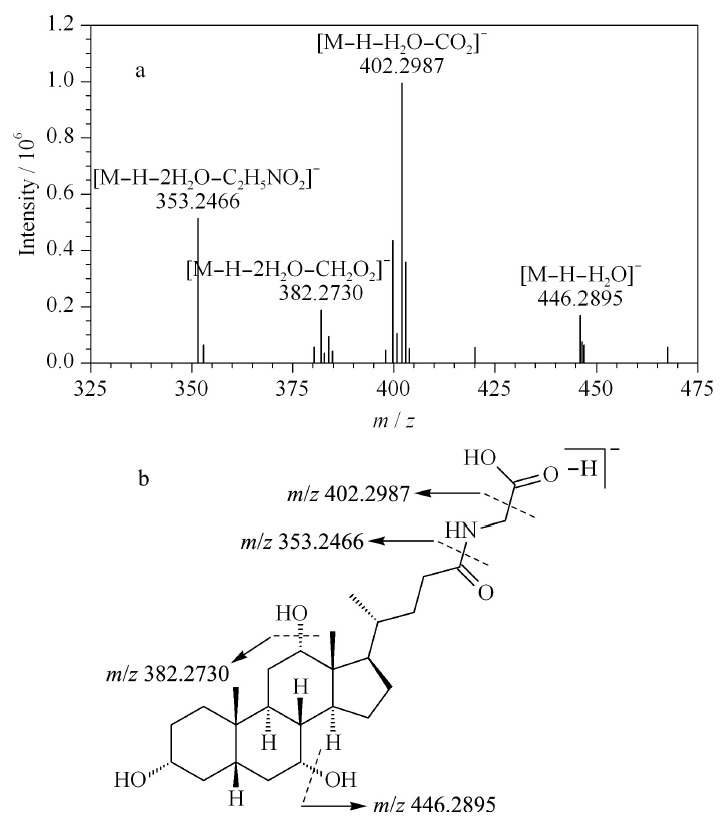
甘氨胆酸的(a)MS^2^图谱和(b)质谱裂解途径

综上,游离型胆酸和结合型胆酸在负离子模式下可得到[M-H]^-^或[M+HCOO]^-^的准分子离子峰。由于甾体母核上存在多个羟基取代,在MS^2^中可生成一定数目的失水离子[M-H-*n*H_2_O]^-^。游离胆酸及甘氨酸结合型胆酸结构中均存在羧基,故会脱羧失去CO_2_(44 Da)。结合型胆酸均由胆酸与牛磺酸和甘氨酸经酰胺键连接,故会生成相应的丢失牛磺酸残基与甘氨酸残基的碎片[M-C_2_H_6_NO_3_S]^-^和[M-C_2_H_4_NO_2_]^-^。本实验所采用的IT-TOF-MS在CID模式下采集数据,并未发现低质量数的牛磺酸结合型以及甘氨酸结合型的特征碎片,如*m/z* 80(S $O_{3}^{-}$ )、124(C_2_H_6_NO_3_S^-^)以及74(C_2_H_4_N $O_{2}^{-}$ ),而且大部分的甾体母核在检测条件下并未发生碎裂。

### 2.4 片仔癀化学成分表征

本工作采用online PLE-UHPLC-IT-TOF-MS方法在负离子模式下检测片仔癀样品,其基峰色谱图见图2a。通过主要成分皂苷以及胆酸类化合物的质谱行为总结,结合对照品以及相关文献,最终从片仔癀中鉴定了71个化学成分,仍有2个化合物的结构尚未确认。通过与对照品比对,化合物9、13、44、45、58、61、65、70、42、50、55、64、66和68分别鉴定为三七皂苷R1、人参皂苷Rf、Rd、Re、Rg2、F2、Rg3、Rh2、GCA、TCDCA、CA、UDCA、HDCA和CDCA。结合参考文献^[12,13]^及裂解途径,从片仔癀中初步鉴定了35个皂苷类化合物和34个胆酸类化合物。对比自建的三七、蛇胆、牛黄和麝香的化学成分数据库,对鉴定的成分进行归属,其中三七来源化学成分36个,包括1个炔的脂肪酸苷(化合物1)以及35个皂苷(化合物2~13、18、19、22、23、31、33~36、43~47、49、54、56、58、59、61、62、65和70),蛇胆来源化学成分15个,均为胆汁酸类化合物(化合物14、15、16、17、20、21、24、25、26、32、37、38、40、41和53),牛黄来源化学成分9个,为化合物51、57、63、64、66、67、68、69和71,牛黄和蛇胆共有成分11个,为化合物27、28、29、30、39、42、48、50、52、55和60(见表1)。

**表 1:** 片仔癀的化学成分信息表

No.Source	*t*_R_/minRef.	Adduct ion	MS^1^(*m/z*)	Formula	Error/10^-6^	MS^2^(*m/z*)	Putative identity
1PN	5.52[14]	[M+HCOO]^-^	549.1841	C_22_H_32_O_13_	2.91	503.1850, 341.1057, 179.0582	notoginsenic acid *β*-sophoroside^#^
2PN	5.87[15]	[M+HCOO]^-^	861.4828	C_42_H_72_O_15_	-2.90	653.4138	notoginsenoside SP1^#^
3PN	6.68[16]	[M+2HCOO]^2-^	592.2883	C_53_H_90_O_23_	-5.91	569.2781	yesanchinoside-H
4PN	6.89[14]	[M+HCOO]^-^	1007.5416	C_48_H_82_O_19_	-1.59	961.5339, 799.4840, 637.4307, 475.3812	notoginsenoside R3/notoginsenoside R6/20-*O*-glucoginsenoside Rf
5PN	7.17[14]	[M+HCOO]^-^	879.5001	C_42_H_74_O_16_	4.78	833.4832, 785.7090, 671.4084	notoginsenoside J/isomer^#^
6PN	7.24[14]	[M+HCOO]^-^	1007.5425	C_48_H_82_O_19_	-0.69	961.5343, 799.4841, 637.4272, 475.3674	notoginsenoside R3/notoginsenoside R6/20-*O*-glucoginsenoside Rf
7PN	7.31[14]	[M+HCOO]^-^	879.5001	C_42_H_74_O_16_	4.78	833.4832, 785.7090, 671.4084	notoginsenoside J/isomer^#^
8PN	7.48[14]	[M+HCOO]^-^	977.5323	C_48_H_82_O_20_	-0.41	931.5145	notoginsenoside ST-5
9PN	7.52[14]	[M-H]^-^	931.5273	C_47_H_80_O_18_	0.11	799.4784, 769.4697, 637.4238, 475.3735	notoginsenoside R1^*^
10PN	7.57[14]	[M-H]^-^	931.5273	C_47_H_80_O_18_	0.32	799.4763, 637.4263	notoginsenoside R1 isomer
11PN	7.68[17]	[M+HCOO]^-^	991.5496	C_48_H_82_O_18_	1.31	945.5354, 783.4815, 621.4317, 459.3765	notoginsenoside K/isomer
12PN	7.76[14]	[M+HCOO]^-^	845.4865	C_42_H_72_O_14_	-4.61	799.4821, 637.4260, 475.3742	ginsenoside Rg1^#^
13PN	7.85-	[M+HCOO]^-^	845.4950	C_42_H_72_O_14_	5.44	799.4773, 637.4278, 475.3748	ginsenoside Rf^*^
14SG	8.06[18]	[M-H]^-^	530.2765	C_26_H_45_NO_8_S	-5.66	512.2659	tauro-3*α*, 7*α*, 12*α*, 23R-tetrahydroxy-5*β*-cholenoic acid/isomer
15SG	8.11[18]	[M-H]^-^	530.2776	C_26_H_45_NO_8_S	-3.21	512.2659	tauro-3*α*, 7*α*, 12*α*, 23R-tetrahydroxy-5*β*-cholenoic acid/isomer
16SG	8.39[18]	[M-H]^-^	530.2762	C_26_H_45_NO_8_S	-5.85	512.2738	tauro-3*α*, 7*α*, 12*α*, 23R-tetrahydroxy-5*β*-cholenoic acid/isomer
17SG	8.48[18]	[M-H]^-^	512.2667	C_26_H_43_NO_7_S	-4.29	480.2223, 456.2491, 358.1651	tauro-Δ8-3*β*, 7*α*, 12*α*-trihydroxy-5*β*-cholenoic acid/isomer
18PN	8.50[16]	[M+HCOO]^-^	815.4782	C_41_H_70_O_13_	-1.96	769.4655, 637.4238, 475.3735	notoginsenoside R2/pseudoginsenoside RT3/isomer
19PN	8.55[14]	[M-H]^-^	1239.6423	C_59_H_100_O_27_	3.55	945.5369, 783.4814, 459.3795	notoginsenoside Ra3/ginsenoside R4/notoginsenoside Fa
20SG	8.60[18]	[M-H]^-^	530.2766	C_26_H_45_NO_8_S	-5.28	512.2660	tauro-3*α*, 7*α*, 12*α*, 23R-tetrahydroxy-5*β*-cholenoic acid/isomer
21SG	8.64[18]	[M-H]^-^	512.2674	C_26_H_43_NO_7_S	-2.54	494.2202, 387.2546, 369.2400	tauro-Δ8-3*β*, 7*α*, 12*α*-trihydroxy-5*β*-cholenoic acid/isomer
22PN	8.71[16]	[M-H]^-^	1239.6360	C_59_H_100_O_27_	-1.1	945.5367, 783.4932, 459.3841	ginsenoside Ra3/notoginsenoside R4/notoginsenoside Fa^#^
23PN	8.78[16]	[M-H]^-^	1239.6407	C_59_H_100_O_27_	2.26	945.5398, 783.4816, 459.3772	ginsenoside Ra3/notoginsenoside R4/notoginsenoside Fa^#^
24SG	8.99-	[M-H]^-^	423.2731	C_24_H_40_O_6_	-2.1	405.2617, 387.2546, 359.2597, 325.2513	3*α*, 6*β*, 7*α*, 12*α*-tetrahydroxy bile acid/isomer
25SG	9.09[18]	[M-H]^-^	530.2767	C_26_H_45_NO_8_S	-5.09	512.2635, 476.2432	tauro-3*α*, 7*α*, 12*α*, 23R-tetrahydroxy-5*β*-cholenoic acid/isomer
26SG	9.27[18]	[M-H]^-^	512.2666	C_26_H_43_NO_7_S	-4.10	456.2306, 358.1544, 328.1518	tauro-3*α*, 7*α*-dihydroxy-12-oxo-5*β*-cholenoic acid/isomer
27BC/SG	9.58[19]	[M-H]^-^	514.2820	C_26_H_45_NO_7_S	-4.67	496.2716, 480.2223, 358.1647, 353.2382	taurocholic acid/tauro-3*α*, 7*α*, 12*α*-trihydroxy-5*α*-cholenoic acid/isomer
28BC/SG	10.00[18]	[M-H]^-^	514.2827	C_26_H_45_NO_7_S	-3.31	496.2707, 353.2382, 329.2513	taurocholic acid/tauro-3*α*, 7*α*, 12*α*-trihydroxy-5*α*-cholenoic acid/isomer
29BC/SG	10.42[20]	[M-H]^-^	514.2846	C_26_H_45_NO_7_S	0.39	496.2722, 353.2466, 329.2496	taurocholic acid/tauro-3*α*, 7*α*, 12*α*-trihydroxy-5*α*-cholenoic acid/isomer
30BC/SG	10.84[21]	[M-H]^-^	514.2825	C_26_H_45_NO_7_S	-3.89	496.2642, 353.2453	taurocholic acid/tauro-3*α*, 7*α*, 12*α*-trihydroxy-5*α*-cholenoic acid/isomer
31PN	11.47[14]	[M+HCOO]^-^	947.5239	C_46_H_78_O_17_	1.90	901.5056	chikusetsusaponin L5
32SG	12.52[18]	[M-H]^-^	512.2686	C_26_H_43_NO_7_S	-0.2	456.2306, 358.1544, 328.1518	tauro-3*α*, 7*α*-dihydroxy-12-oxo-5*β*-cholenoic acid/isomer
33PN	13.01[14]	[M-H]^-^	1107.5913	C_54_H_92_O_23_	-3.97	945.5398, 783.4833, 765.4795	ginsenoside Rb1^#^
34PN	13.40[14]	[M-H]^-^	1107.5999	C_54_H_92_O_23_	3.79	945.5380, 783.4814, 765.4736	yesanchinoside-E^#^
35PN	13.75[14]	[M+HCOO]^-^	815.4796	C_41_H_70_O_13_	-0.25	769.4657, 637.4254, 475.3859	notoginsenoside R2/pseudoginsenoside RT3/isomer
36PN	15.06[14]	[M+HCOO]^-^	815.4758	C_41_H_70_O_13_	-4.91	769.4657, 637.4252, 475.3665	notoginsenoside R2/pseudoginsenoside RT3/isomer
37SG	15.55[18]	[M-H]^-^	530.2770	C_26_H_45_NO_8_S	-4.34	512.2633	tauro-3*α*, 7*α*, 12*α*, 23R-tetrahydroxy-5*β*-cholenoic acid/isomer
38SG	16.13-	[M-H]^-^	423.2740	C_24_H_40_O_6_	-2.84	405.2602, 325.2517	3*α*, 6*β*, 7*α*, 12*α*-tetrahydroxy bile acid/isomer
39BC/SG	17.11[21]	[M-H]^-^	405.2623	C_24_H_38_O_5_	-5.68	359.2492, 343.2637	3*α*, 12*α*-dihydroxy-7-oxo-5*β*-cholic acid/isomer
40SG	17.79-	[M-H]^-^	423.2737	C_24_H_40_O_6_	-9.21	405.2613, 325.2517	3*α*, 6*β*, 7*α*, 12*α*-tetrahydroxy bile acid/isomer
41SG	18.58-	[M-H]^-^	423.2731	C_24_H_40_O_6_	-7.80	405.2673, 325.2513	3*α*, 6*β*, 7*α*, 12*α*-tetrahydroxy bile acid/isomer
42BC/SG	18.98-	[M-H]^-^	464.3001	C_26_H_43_NO_6_	-3.66	446.2895, 402.2987, 382.2730, 353.2466	glycocholic acid^*^
43PN	19.31[17]	[M+HCOO]^-^	991.5481	C_48_H_82_O_18_	-0.20	945.5323, 851.5223, 726.4848, 673.5301	gypeniside VIII
44PN	19.89-	[M+HCOO]^-^	991.5483	C_48_H_82_O_18_	0.00	945.5388, 783.4813, 621.4303, 459.3799	ginsenoside Rd^*^
45PN	20.03-	[M-H]^-^	945.5457	C_48_H_82_O_18_	3.07	783.4815, 765.4795, 621.4319, 459.3767	ginsenoside Re^*^
46PN	20.47[14]	[M+HCOO]^-^	683.4354	C_36_H_62_O_9_	-3.22	637.4279, 475.3736	ginsenoside Rh1/isomer^#^
47PN	21.57[14]	[M-H]^-^	945.5458	C_48_H_82_O_18_	3.17	783.4807, 621.4295, 459.3835	notoginsenoside K/isomer
48BC/SG	22.34[21]	[M-H]^-^	405.2607	C_24_H_38_O_5_	-9.62	343.2625, 289.2152, 251.1989	3*α*, 12*α*-dihydroxy-7-oxo-5*β*-cholic acid/isomer
49PN	22.95[14]	[M+HCOO]^-^	683.4376	C_36_H_62_O_9_	0.00	637.4263, 475.3761	ginsenoside Rh1/isomer^#^
50BC/SG	23.48[17]	[M-H]^-^	498.2881	C_26_H_45_NO_6_S	-2.81	480.2806, 355.2612	taurochenodeoxycholic acid^*^
51BC	24.42[17]	[M-H]^-^	498.2879	C_26_H_45_NO_6_S	-3.21	480.2756, 355.2607	taurodeoxycholic acid^#^
52BC/SG	25.56[21]	[M-H]^-^	405.2633	C_24_H_38_O_5_	-3.21	343.2632, 289.2158, 251.1991	3*α*, 12*α*-dihydroxy-7-oxo-5*β*-cholic acid/isomer
53SG	25.82-	[M-H]^-^	487.2368	C_24_H_40_O_8_S	0.82	452.1676, 408.2813	cholic acid-sulfate
54PN	26.05[14]	[M-H]^-^	915.5297	C_47_H_80_O_17_	-2.84	-	gypeniside IX
55BC/SG	26.79-	[M-H]^-^	407.2785	C_24_H_40_O_5_	-4.42	389.2666, 361.2698, 345.2771, 343.2618, 327.2652	cholic acid^*^
56PN	27.16[14]	[M+HCOO]^-^	815.4796	C_41_H_70_O_13_	-0.25	769.4657, 637.4307, 475.3812	notoginsenoside R2/pseudoginsenoside RT3/isomer
57BC	29.50[22]	[M-H]^-^	448.3042	C_26_H_43_NO_5_	-5.80	404.3812, 386.3058, 355.2611	glycochenodeoxycholic acid
58PN	30.95-	[M+HCOO]^-^	829.4933	C_42_H_72_O_13_	-2.65	783.4795, 637.4115, 475.3675	ginsenoside Rg2^*^
59PN	31.67[14]	[M+HCOO]^-^	829.4918	C_42_H_72_O_13_	-4.46	783.4793, 621.4165, 459.3762	ginsenoside Rg3 isomer
60BC/SG	32.56[22]	[M-H]^-^	448.3054	C_26_H_43_NO_5_	-3.12	430.2918, 402.2990, 386.3048	glycodeoxycholic acid^#^
61PN	33.96-	[M+HCOO]^-^	829.4933	C_42_H_72_O_13_	-2.65	783.4795, 621.4337	ginsenoside F2^*^
62PN	36.57[14]	[M-H]^-^	665.4277	C_37_H_62_O_10_	1.05	-	notoginsenoside T2/isomer
63BC	37.39-	[M-H]^-^	465.3209	C_27_H_46_O_6_	2.79	401.3024, 383.2961, 263.1984	tetrahydroxycholestan-26-oic acid^#^
64BC	38.65-	[M-H]^-^	391.2815	C_24_H_40_O_4_	1.20	345.2769, 327.2698	ursodeoxycholic acid^*^
65PN	39.23[14]	[M+HCOO]^-^	829.4974	C_42_H_72_O_13_	2.29	783.4743, 621.4149, 459.3672	ginsenoside Rg3^*^
66BC	40.56-	[M-H]^-^	391.2838	C_24_H_40_O_4_	-4.09	345.2788, 327.2657	hyodeoxycholic acid^*^
67BC	40.76-	[M-H]^-^	389.2681	C_24_H_38_O_4_	-4.11	371.2567, 309.2194	ketodeoxycholic acid^#^
68BC	41.52-	[M-H]^-^	391.2838	C_24_H_40_O_4_	-4.09	373.2574	chenodeoxycholic acid^*^
69BC	42.50-	[M-H]^-^	391.2838	C_24_H_40_O_4_	-4.09	345.2792, 327.2700	deoxycholic acid^#^
70PN	43.57[14]	[M+HCOO]^-^	667.4365	C_36_H_62_O_8_	-9.29	621.4281, 459.3800	ginsenoside Rh2^*^
71BC	44.24-	[M-H]^-^	421.2950	C_25_H_42_O_5_	-2.14	375.2884, 273.2215	methyl cholate
72N. A.	44.62-	[M-H]^-^	299.2565	C_18_H_36_O_3_	-8.35	281.2453, 253.2515, 225.2123	unknown
73N. A.	45.29-	[M-H]^-^	795.5396	C_43_H_76_N_2_O	-2.51	405.2581, 389.2588, 343.2570	unknown

Note: PN, Panax Notoginseng; BC, Bovis Calculus; SG, Snake Gall; * confirmed with authentic compounds; # inferred from mass fragmentation pathways; N. A., not applicable.

### 2.5 讨论

中药常用的提取方法,如连续回流法、超声提取法等,需要大量的溶剂和药材,费时费力,且不适用于贵细中药材的分析。本文建立了在线加压溶剂提取方法,采用水作为绿色提取溶剂,通过升温和加压改变水的理化性质,提高水对药材中化学成分的溶解和提取能力。同时,本法提取效率高,仅需微量样品,提取3 min,可提取出中药复方中丰富的化学成分。

通过质谱检测发现片仔癀的主要化学成分类型为皂苷和胆酸,且经各已知单味药化学成分数据库比对,皂苷多来源于中药三七,胆酸类主要来源于牛黄和蛇胆,由于麝香的化学成分主要为大分子环酮及胆甾醇等小极性化合物,本方法并未能检出该类成分,后续将利用气相色谱或超临界流体色谱-串联质谱等技术对其进行检测和鉴定。

## 3 结论

本文采用online PLE-UHPLC-IT-TOF-MS法对片仔癀成分组进行快速全面分析,为其质量控制研究提供可靠的依据。通过对照品比对、质谱裂解规律推断以及相关文献参考,共鉴定71个化学成分,推测其中36个化合物来源于三七,15个化合物来源于蛇胆,9个化合物来源于牛黄,11个为牛黄与蛇胆来源成分。Online PLE-UHPLC-IT-TOF-MS法不仅简化了样品前处理步骤,节省了溶剂和人力,而且能够实现中药复方制剂的直接分析,践行了绿色化学的理念,提高了分析的准确度和灵敏度。
